# The missing piece. The perceived marginalization of CSOs and HRGs in SOSTs’ decision-making processes: a review

**DOI:** 10.12688/openreseurope.15078.2

**Published:** 2023-06-27

**Authors:** Lorenzo De Sabbata, Riccardo Laterza, Daniele Del Bianco, Olivia Ferrari, Ezio Benedetti

**Affiliations:** 1 Institute of International Sociology of Gorizia – ISIG, Gorizia, Italy, 34170, Italy; 2 Department of Social and Political Sciences, University of Trieste, Trieste, 34127, Italy

**Keywords:** participation, border surveillance, participatory models, Surveillance-Oriented Security Technologies (SOSTs) for borders control, citizens’ involvement, privacy, fundamental rights

## Abstract

This review represents the first contribution of a research diptych which stems from the activities implemented in the framework of the H2020 ARESIBO project (Augmented Reality Enriched Situation awareness for Border security). The general objective of ARESIBO is to improve the efficiency of border surveillance systems by providing the operational teams, as well as the tactical command and control level with accurate and comprehensive information related to border control considering different issues and perspectives. These perspectives also include the analysis of the level of engagement and the (possible) enhancement of citizens’ involvement in the development and decision making related to border surveillance.

The principal human rights and migration International Organisations (IOs), as well as EU institutions dealing with security and external borders (i.e., Frontex), agree and state that human rights groups (HRGs) and civil society organisations (CSOs) should be more involved and integrated in border surveillance. That stated, the main goal of this paper is to analyse this perceived marginalisation of CSOs and HRGs, as it emerges from several HRGs’ and CSOs’ statements on that regard in order to explore the reasons of this perceived marginalisation, as well as the elements that on the contrary seem to make this marginalisation less substantial. The results of this non-systematic review in the framework of ARESIBO led to the elaboration of an innovative participatory model that will be analysed in detail in the second article of this research diptych, entitled “Towards the engagement of citizens in SOSTs decision-making: participatory models setting a common ground for border surveillance and respect of fundamental rights. The case of ARESIBO H2020 project”.

## Introduction

### The importance of a human rights-based approach in border management and border surveillance

Scholars and international organizations (including the EU and its agency dealing with the management of external borders – Frontex ^1^) agree that an enhanced and more structured involvement of Civil Society Organisations (CSOs) and Human Rights Groups (HRGs) in decision making related border management and surveillance is of paramount importance to guarantee a consistent application and respect of fundamental rights, especially when the use of new technologies (e.g., use of AI in border surveillance, collection of biometric data at border crossings, etc.) is foreseen ( McAdam, 2013).

For instance, the UN Office of the High Commissioner for Human Rights (OHCHR) affirms that: « *States have legitimate interests in implementing border controls, in order to enhance security, to protect human rights, and to respond to transnational organized crime*», […] and « *assert a human rights-based approach deriving from the core international human rights instruments and anchored in the interdependence and inalienability of all human rights, seek to establish accountability between duty-bearers and rightsholders, emphasis participation and empowerment, and focus on vulnerability, marginalization and exclusion.*» ( OHCHR, 2014, 12).

The need (and the request) to ensure citizens’ and States’ security favoured the development and the introduction of new technologies and new approaches to border controls. In this frame, screening procedures at borders are deemed to play an important role in guaranteeing the overall security of the country, in particular in its efforts to fight against potential terrorist acts. At the same time, States are bound by international law to respect and protect the human rights of every individual while establishing security the measures and applying innovative and/or technological measures to ensure the control and the management of borders.

Thus, when dealing with border security and border management, it is not possible to disregard and underestimate the impact that counter terrorism measures and the adoption of SOSTs at borders have or may have on the fundamental rights of individuals, who are entitled to cross borders under determined conditions, such as economic migrants, individuals that ask for international protection – such as refugees and asylum seekers – as well as normal citizens who are travelling form a country to another for work or leisure.

Despite this general institutional and academic consensus, several HRGs and CSOs working in the framework of border surveillance issues perceive to be marginalised from the overall national and European border management system, and report(ed) and denounce(d) this marginalisation at different levels. The first goal of the paper is thus to explore this perceived marginalisation, giving an account on the main reasons for it, as they are expressed by HRGs and CSOs. At the same time, this review takes into account and outlines the ways in which HRGs and CSOs are included in border management, focusing in particular on FRONTEX, from the point of view of legislation and of its consultancy bodies. With these aims, following the focus of the ARESIBO project, the paper focuses in particular on the Mediterranean Region, with some references to the Hungarian-Serbian border. In its conclusions, the paper introduces the ARESIBO Participatory Model (APM), a participatory methodology elaborated in the frame of the project ARESIBO as a proposal to enhance participation of HRGs and CSOs in the development of border surveillance technologies, thus addressing this perceived marginalisation in a more consistent and structured manner ^2^.

### What is the role of HRGs and CSOs in border management and in SOSTs decision making?

The analysis of the perceived marginalization of HRGs and CSOs in decision making related to border surveillance and border management stems from the consideration that many HRGs and CSOs have reported and criticized several human rights violations in recent years, including the rejection (i.e., *refoulement ^3^*) of migrants and asylum seekers that involved border surveillance and management methods. Additionally, HRGs have asserted that they are being more and more excluded from discussions and decisions on these matters. According to such viewpoints, a security-focused, profit-driven, and technology-based approach to the 3mportanti replacing one that prioritizes human rights, portraying migration as primarily a security threat and technology as the primary means to address it.

Although it might be viewed as being somewhat essentialist, this dichotomy is a useful theoretical tool for evaluating and analysing in a broader context the various positions taken by HRGs and CSOs regarding border control technologies and how those positions may interact with the defence of immigrants’ and asylum seekers’ human rights as well as the rights of European citizens. The significant points made by various HRGs and CSOs in response to three primary research questions are summarized in this section.

The three research questions are the following. How do HRGs and CSOs describe the alleged marginalization process and which role do these stakeholders play in Europe’s public and political discourse on that regard? Second, what are the major opinions about the platforms for engagement and points of contact between EU institutions and HRGs/CSOs? Third, what is the dialectic existing between the issues of respect of human rights and the articulated and complex system of procedures and rules put in place to enforce border controls in the EU?

Following this approach, to set the ground for the in-depth analysis of perceived marginalization of HRGs and CSOs in relation to border surveillance and border management policy making, the paper first provides for the results of a) a press review on EU relevant public debates involving the issues of border control and human rights (par. 3); b) a review of the integration of Protection of Fundamental Rights in Frontex legal basis, guidelines and code of conduct (par. 4). Second, the review focuses on the progressive creation of a “border-industrial complex” in Europe (par. 5), as some HRGs put it when criticizing what they perceived to be as an increased political and financial influence of industrial security lobbies on Frontex ^4^ politics at the expense of a more human rights-oriented approach. Finally, the review focuses on how participatory tools used by EU institutions to consult HRGs and CSOs on migration policies and border management/surveillance have been received and applied (par. 7), and on the main criticisms moved by CSOs and HRGs regarding these processes, focusing in particular on the case of Frontex’s Consultative Forum on Human Rights (par. 6).

### Border management, HRGs/CSOs and human rights: a press review

Euro|topics ^5^ served as the main tool for the press-review analysis realised for this contribute, in the framework of ARESIBO. Since 2005, euro|topics offers data on more than 500 print and online publications from more than 30 nations, including every EU member state.

From a methodological standpoint, the portal was searched for using both the terms “border” and “human rights”, focusing on the timeframe 2019–2021. Results that were neither directly nor indirectly related to EU member states or EU institutions, as well as articles that focused on more general geopolitical issues, were not considered. Covid-19-related articles were also removed from the list. This decision has been taken to avoid an excessive focus on the pandemic circumstance and its impact on borders’ management (precisely because of the exceptional nature of the situation).

The review included 51 materials that directly or indirectly address human rights concerns in relation to border security. The selection has been based on 10 debates included in articles published on relevant European newspapers in 2019–2021 ^6^. According to the 10 selected debates, the 51 newspaper articles are distributed as follows:

1. Cases of sexual abuse of children by refugees or asylum seekers in Finland (1 article).
2. The Central Mediterranean migrant crisis which stood at the centre of a debate among EU member states on migrant rescue and re-distribution, in Summer 2019, and again in Spring-Summer 2020 (12 articles).
3. The presentation of the new EU Commission by President Ursula von der Leyen, in September 2019 (3 articles).
4. 39 dead bodies of migrants found in a lorry nearby London, in October 2019 (4 articles);
5. The Aegean Sea migrant crisis involving the island of Lesbos, Chios and Samos, which experienced a huge pressure with the installation of overcrowded refugee camps, between Autumn 2019 and Winter 2020 (4 articles);
6. The January 2020 ECHR ruling that gave Spain permission to continue pushing back undocumented migrants from Ceuta and Melilla to Morocco, a procedure known as "hot return" (3 articles);
7. The 2020 migrant crisis on the border between Greece-Turkey (19 articles);
8. The massive fire that destroyed the Moira camp in Greece in September 2020, which was built to house 2,800 people but was occupied at the time by 12,000 asylum seekers (1 article);
9. The New EU Asylum Pact presented by the EU Commission in September 2020, few weeks after the Moira Fire (2 articles);
10. The response to media reports from December 2020 that claimed Frontex was personally responsible for unlawful pushing back of immigrant crossings at the Greek sea border (2 articles).

The selected articles provide a variety of viewpoints, which can be grouped, analysed, and classified in accordance with opinions on EU border control and immigration policy, using two criteria. First, the fairness of implemented policies considering human rights and, more broadly, EU fundamental principles. Second, the effectiveness of imposed restrictions in comparison to illegal immigration and human trafficking, as one of the national and EU interests that must be upheld.

In this perspective, a relevant majority of articles – 31 out of 51 – put into question the fairness of EU policies, measures, and/or practices in border and migration control, while 19 out of 51 articles address the effectiveness of those same policies, measures, and practices. 11 out of 51 articles address both the fairness and the efficacy of these policies, measurements, and/or practices, whereas only 2 out of the 11 articles state that they are both unfair and ineffective. The approach to EU border management is not presented as fair but ineffective in any of the articles ^7^.

In addition, two relevant elements are present in all the article excerpts, both relevant for the purposes of this review. First, considerations and/or criticisms on the use of technology to accomplish the objectives associated to border control remain in the background. Policies and practices that make use of such technology to achieve certain border management goals seem to be the main issue at stake from the perspectives of both “realist” – i.e., those who believe border policies are effective – and “critical”– i.e., those who believe border policies are unjust – observers. In other words, it seems that mainstream media do not cover in-depth examinations of applied technologies.

The second 5mportanti that, even though the term “human rights” is mentioned in more than one article, along with references to the 1951 Geneva Convention ^8^ and the European Convention on Human Rights (ECHR) ^9^, there is no direct mention of specific HRGs or CSOs in the selected articles.

### The role of Frontex for the protection of human rights: legal framework, guidelines and Code of Conduct

For what concerns Frontex and its role towards the protection of human rights, Hruschka (2020) affirms that the fundamental objective of Frontex – « *to eliminate irregular border crossings of the external borders*» – is in constant conflict with « *a human rights-based approach to the European migrant policy*» ( Hruschka, 2020). Over time, this friction caused various public scandals, and the Agency frequently came under criticism from political groups, CSOs and HRGs.

However, the respect and the commitment for fundamental rights has been a part of Frontex’s mandate since its origins. The review of the primary legislative provisions that form the Agency’s legal basis demonstrates how increasingly important the preservation of basic rights has become to Frontex during these last few years. The goal of this section is to highlight that a human rights-based approach is enshrined in the legal provisions that created and regulate the work of the Agency, even if it is not always possible to assess consistently how these principles and rules are effectively applied in border management.

Following this approach, this review analysed the four (4) main documents which constitute the legal basis of the Agency’s action and mandate ^10^. These four documents will be briefly recalled hereinafter in relation to their relevance to the human rights’ dimension.

The first 6mportanti the Regulation No. 2007/2004 of 26 October 2004. This Regulation of the EU is the first legal cornerstone for Frontex. The document refers to the respect and protection of fundamental rights in a very limited manner, with only Recital 22 mentioning it, as follows: « *This Regulation respects the fundamental rights and observes the principles recognised by article 6(2) of the Treaty on European Union and reflected in the Charter of Fundamental Rights of the European Union*».

In this sense, it may be possible to argue that this reference to the fundamental principles governing the action of the EU is more a technicality and a legal duty than a real commitment enshrined in the Agency’s mandate. However, in the next document considered for this article, Regulation No 1168/2011, which is an amendment to the Regulation No. 2007/2004, references to fundamental rights increase significantly. Article 1 of this regulation suggests that the « *development of a forward-looking and comprehensive European migration policy, based on human rights, solidarity and responsibility, especially for those Member States facing specific and disproportionate pressures, remains a key policy objective for the Union*». It must be noted also that this document refers to “fundamental rights” over 35 times. Notably, such references include the broad contours of Frontex's mandate as well as several distinct areas of the Agency’s activity, including refugees and asylum seekers, staff training, and collaboration with third countries ^11^.

A further evolution and increased attention to the fundamental rights dimension has been recorded in 2016 with the adoption and entry in force of Regulation No 2016/1624. References to “fundamental rights” are present in this document over 100 times and they address the general framework of Frontex’s mandate in a specific manner. More in detail, art. 2 strengthens the role of fundamental rights in the Agency’s overall mandate ^12^; art. 14 rebalances the weight of fundamental rights in Frontex’s tasks and competences ^13^; and, for the first time ever since Frontex exists, art. 47 lists in a consistent and comprehensive manner the main fundamental rights references and international conventions guiding the Agency’s action ^14^.

Finally, there are almost 200 instances of “fundamental rights” mentioned in the most recent legal act devoted to Frontex, the Regulation 2019/1896. These references cover the broad contours of Frontex’s mandate as well as several distinct areas of the Agency’s activity, including administration of hotspot regions, staff training, return decisions and operations, collaboration with foreign countries, and development of a complaint mechanism ^15^, which can be identified as the most innovative part of the document for what concerns human rights.

It is therefore possible to affirm that Frontex’s legal basis clearly enshrines fundamental rights and freedoms in its mandate. Moreover, the Agency strictly adheres to the Charter of Fundamental Rights of the EU, the European Convention for the Protection of Human Rights and Fundamental Freedoms, as well as relevant international law. It is, however, more difficult to ascertain how these guiding principles are then translated into action.

In this perspective, it seems relevant to focus on two documents that regulate the Agency’s actions: Frontex’s Fundamental Rights Strategy and Code of Conduct. Since 2012, Frontex’s Fundamental Rights Officer oversees the drafting of the Agency’s Fundamental Rights Strategy (as foresaw by the Regulation 1168/2011 and confirmed in the Regulation 2019/1896) to integrate fundamental rights’ respect and protection in the performance of its daily tasks. The Strategy is implemented within the Agency’s Action Plan, and it specifies practical fundamental rights protections that direct the Agency's operational efforts in the direction of the accomplishment of its mission and operational objectives. Frontex openly disseminates among the pertinent national, European, and/or international entities the goals and content of its Strategy, aiming to increase the transparency of its operations in the process.

The Frontex Strategy for 2021 has been developed in accordance with 9 principles that direct the Agency’s actions and conduct with regard to border surveillance and the management of migratory flows. Here is a synthetic overview of such principles according to the Agency’s Fundamental Rights Strategy endorsed by the Fundamental Rights Officer on 25 January 2021 and adopted by the Management Board on 14 February 2021: « *1. Right to seek asylum. 2. Attention to the needs of vulnerable persons or groups and persons in a vulnerable situation, including children. 3. Best interests of the child as a primary consideration when taking any decisions affecting children. 4. Equality and non-discrimination in the design and implementation of border management measures. 5. Gender equality as an integral part of their work and resolve to include gender considerations throughout their operational activities. 6. Accountability of the agency to the European parliament and to the council as well as to the court of justice of the European union. 7. Transparency, good administration and access to documents held or produced by the agency. 8. Fundamental rights due diligence to all agency’s activities, ensuring the highest standard of performance, assessing and mitigating the risk of violating fundamental rights from planning through monitoring and evaluation. 9. Protection of personal data*» (pp. 5–6).

The Strategy 7mportanti s7 a section dedicated to the state-of-the-art in the use of technology and in the development of SOSTs in border surveillance that may improve European Integrated Border Management and ensure Frontex’s compliance with fundamental rights. In this perspective, the document states that « *Compliance with fundamental rights, including the protection of personal data and non-discrimination, shall be a core element in the establishment and running of large-scale information systems under the responsibility of the Agency*», stressing that « *coherent approach to the strategic and ethical aspects of sustainability and social responsibility is a key factor*» (p. 12) in this regard ^16^.

As mentioned before in this paragraph, the 2011 amendment of the Frontex system included in Regulation 1168/2011 also added the requirement for the Agency to create a Code of Conduct that lays out « *procedures intended to guarantee the principles of the rule of law and respect for fundamental rights with particular focus on unaccompanied minors and vulnerable persons, as well as on persons seeking international protection, applicable to all persons participating in the activities of the Agency* » (art. 2°). The Agency’s *Code of Conduct for all persons participating in Frontex operational activities* is conceived to help « *demonstrate the commitment made by Frontex to promote and enforce the highest professional and behavioural standards during operational activities. Such a commitment extends to fully respecting, upholding and fulfilling fundamental rights*» (p. 5). There are seven articles of the *Code of Conduct* that might be considered as most relevant when it comes to the promotion and protection of fundamental rights by the Agency and its personnel.

The first section of the *Code* considering fundamental freedoms and human rights is art. 4. This article provides for the promotion, respect and fulfilment of fundamental rights for all individuals « *regardless of their sex, race, colour, ethnic or social origin, genetic features, language, religion or belief, nationality, political or any other opinion, membership of a national minority, property, birth, disability, age or sexual orientation or other status, with particular focus on vulnerable persons*» and it « *ensure respect for the relevant international and European instruments regarding fundamental rights protection, including the asylum acquis».* The second relevant section is included in art. 5, which provides for the respect and fulfilment of the rights of individuals seeking for international protection ^17^ (i.e. asylum seekers, refugees). Thirdly, art. 6 insists on the performance of duties dimension. In fact, it provides for the accountability of the Agency’s staff on fundamental rights in performing their duties. The fourth relevant section is included in art. 9 where the *Code of Conduct* affirms the confidentiality principle in relation with the procession of personal data and the need to comply with EU and national laws on personal data protection. The next two sections are included respectively in art. 10 and art. 12. Art. 10 insists on the need to stick on behavioural standards for the staff employed by the Agency ^18^, while art. 12 provides for the strict application of non-discrimination principle to all activities implemented by Frontex. The seventh, and last, section of the *Code* (art. 20) establishes the duty to cooperate and inform affirming that « *participants must provide information about the complaint mechanism and complaint form to any person who wants to report an alleged fundamental rights violation under that mechanism” (p. 21)*» ^19^.

### 1. HRGs and CSOs vis-à-vis the security-industrial complex

This paragraph focuses on the alleged dependence of Frontex from the “industrial-military complex”, which, according to some HRGs’ and CSOs’ narrative and perception, tends to impose its will and vision on the action of the Agency when it comes to fundamental rights and respect of fundamental freedoms in border controls and management.

 It is rather challenging to put together a comprehensive map of every critical viewpoint HRGs have expressed on border controls and surveillance technologies. In fact, a tremendous number of remarks, inquiries, complaints, allegations, and claims have been made by HRGs and CSOs against the general EU migration policy and the action of Frontex in the last 5 to 10 years. These positions, although frequently have a similar ethical foundation, vary from one another depending on the specific area of influence of each group, which can be territorially or thematically defined. For any of the aforementioned HRGs, such differentiation ensures a particular environment for action as well as areas for self-organization and self-representation. Thus, the goal of this analysis is to concentrate on a specific standpoint that several groups appear to share: the perception of a progressive disregard for fundamental rights concerns and priorities when defining border control policies and deploying surveillance technologies. In other words, HRGs and CSOs actually view themselves as marginalized actors within the framework of border control policy-making, in favour of the emergence of a so-called “ *European security-industrial complex*”.

The term “ *security-industrial complex*” and its conceptual variants recall the earlier “ *military-industrial complex*”, which was used to describe the close connection existing at the time of Cold War between State institutions, their military apparatuses, and the economic sectors supporting them, merging in a potent bundle of interests, in particular in the USA ^20^. The security-industrial complex is accredited to have shifted the same logic on the field of borders control and internal security, at European level. In other words, while criticising the overall structure of EU migration policy, several NGOs, HRGs, and think-tanks maintain that the security and technology sectors are intensifying their lobbying efforts to affect EU policies towards migration in general and Frontex activity in particular.

This theoretical approach is at the same time sustained and sustaining field action and lobbying efforts of HRGs. However, it must be said that such a definition is not matched by the research production at an EU level; by searching the term in the JRC repository, for instance, no results are found ^21^. The definition of security-industrial complex and its conceptual variations – i.e. surveillance-industrial complex – benefits, nevertheless, of a growing attention in academic research and, of course, in the human rights activists’ public debate.

According to researcher Mark Akkerman in an article published in the journal Crisis, the « *creation of the Schengen Area, which coupled the opening of the internal EU borders with robust control at the external borders*» marks the beginning of this trend. He does, however, add that « *especially since the “refugee crisis” of 2015, the EU has accelerated the boosting and militarising of border security, enormously*» ( Akkerman, 2020). The author correlates this process with the increase in the EU budget dedicated for strengthening member states’ border security ( Akkerman, 2020) ^22^. According to the European Parliament Legislative Train 04/32021 IBMF, the instrument for financial support for border management and visas, « *The IBMF would provide funding towards building and enhancing Member States’ capacities in the common visa policy area and in dealing with migratory challenges and potential future threats at external borders, but also in addressing serious cross-border crime and ensuring a high level of internal security within the EU”. According to the European Parliament briefing Migration and border management. Heading 4 of the 2021- 2027 MFF, its budget would amount to “€ 6,5 billion (in 2018 prices) for the 2021–2027 period*», an increase of 135% (p. 5). Additionally, the Multiannual Financial Framework (MFF) foresees a reasonable rise in Frontex funding from 2021 to 2027, « *since 2019 already the third largest traditional EU agency based on the contributions it received*» (p. 5).

Akkerman as well as Douo, Izuzquiza and Silva highlight how these processes are somehow fuelled by the diffusion of a public representation of migration essentially as a threat, a problem that can only be addressed increasing security measures ( Akkerman, 2020; Douo *et al.*, 2021). Most of the critical reviews of EU border control policies and Frontex conduct focus indeed on migration issues. In this perspective, in the report *Guarding the Fortress. The role of Frontex in the militarization and securitisation of migration flows in the European Union* researcher Ainhoa Ruiz Benedicto of the Centre Delàs d’Estudis per la Pau argues that « *population movement is understood and treated as a suspicious activity that needs to be controlled, monitored and registered, while the migration of often forcibly displaced people and refugees is seen as a security threat that must be intercepted*» ( Ruiz Benedicto, 2019, 5).

Following the same perspective, Ruiz argues that the concept of “securitisation” aims to describe a process « *which expands the vulnerability to threats. This means that elements belonging to the sphere of public policy come to be seen as matters of security, which in turn diversifies and generates new threats*» ( Ruiz Benedicto, 2019, 12). Securitizations would create the chance to employ border control in a border context as a technique to identify and screen those elements that, while attempting to cross the border, pose a hazard to the organization in charge of managing the aforementioned border. According to Ruiz, the “securitization approach” in modern European countries mandates the « *technologization of security, wherein security is subordinated to technology*» and « *data-gathering and analysis systems are increasingly consolidated, together with an expansion of the collection of biometric data and its analysis, using algorithms to detect elements of risk*» ( Ruiz Benedicto, 2019, 12). Such perspective on securitisation processes also includes the militarization paradigm, since it conflates the spheres of public safety and war and it integrates military principles in civil matters like borders’ control: « *the use of force and coercion, more weapons equating to more security, and the achievement of security by eliminating threats*» ( Ruiz Benedicto, 2019, 12). According to Akkerman and Ruiz, « *the lobby organisations of large European Commission and EU border agencies*» play a crucial role in pushing the securitization paradigm, which is not just promoted at the political and military levels ( Akkerman, 2020; Ruiz Benedicto, 2019).

In parallel, in their 2017 report *Market forces*, Statewatch and the Transnational Institute (TNI) interpreted the development of EU funded research projects in the field of security as the attempt of building up a strong network capable to orient the whole system towards treating the entire population as potential objects of suspicion ^23^. Such a strong assumption is supported by the description of the “ *European security-industrial complex*” throughout the entire report, in which the establishment of the European Security Research Programme (ESRP) in 2007 assumes a crucial role. Also, Jones claim that a conflict arose around the creation of ESRP between the attempt to develop a “human security” research agenda, as proposed by some actors involved in its development, and the resistance opposed by the traditional setting fuelled by security industry, aimed at directly commercialising industry “solutions” ( Jones, 2017, 15). He affirms that, in the end, the ESRP was designed under the heavy influence of security, defence and technology corporations and research institutes. In his understanding of the issue, this happened at the same time as « *the EU acquired more legislative and financial powers in matters related to the Area of Freedom, Security and Justice’, leading to the acquisition of significant infrastructure for border control and law enforcement and the development of new transnational bodies and networks*» ( Jones, 2017, 15).

Similar findings are included in a 2010 study undertaken for the European Parliament (EP) that highlights that, under the 7 ^th^ Research Framework Programme FP7 2007–2013, « *social science has too often been relegated to a mere ‘ethical’ afterthought, subordinated to concerns with technical deliverables and profit” and that “technological tools and services cannot be developed without a thorough legal, social and political assessment, in order to determine their impact and effects*» ( Bigo *et al.*, 2014). The study argues that « *Security research puts research at the service of industry rather than society. This assumption overrules all other societal considerations, which are relegated to preoccupations with societal acceptance of security technologies*» ( Bigo *et al.*, 2014).

According to the Horizon Europe Strategic Plan 2021–24, that was published by the Directorate–General for Research and Innovation (European Commission), the advisory group’s overall composition has changed over time, to varied degrees adjusting the proportion of industrial and other interests. However, the European Commission now explicitly aims to ensure greater industry involvement in 11mportan strategic direction and the resulting projects.

Hayes describes the interaction between governments, state agencies, and businesses engaged in monitoring as “intimate”, creating a « *perverse political and economic model*» using the concept of « *the surveillance-industrial complex*» ( Hayes, 2012). According to this author, the surveillance-industrial complex is characterized by a blending of internal and external defence lines, which has led to a significant transfer of military logics and technologies into the field of policing and security. This, in turn, has an impact on the requirement to promote interoperability of various security levels and systems ( Hayes, 2012).

But what is the real role of industry lobbies in EU security?

Lemberg-Pedersen and Rübner Hansen affirm that « *the framing of technological border infrastructures as a politically neutral growth area*» promote « *European industrial competitiveness, abstracts from the violent and politically contested character that EU border politics have attained during the last decades*» ( Lemberg-Pedersen & Rübner Hansen, 2020, 48). The authors argue that, in the framework of a EU border market that is multisectoral « *but dominated by the security and defence industry*», the latter influences EU policy-making processes through the organisation and participation in so-called “blurred forums” – roundtables, Commission-initiated expert groups, consultations about research programmes and hardware/software fairs and meetings hosted by industry or public actors – that bring together public policy-makers with the private interest of technology suppliers to shape EU border control priorities and technological capacity ( Lemberg-Pedersen & Rübner Hansen, 2020, 47). The report aims also to show how the same logic affects academia and research, as these institutions are further imbricated in the development of EU border technology through FP7 and H2020 consortiums.

The marginalisation of human rights groups and civil society organisations appears to be as a fundamental characteristic emerging from the arguments proposed by HRGs and scholars who insist on the progressive implementation of a European Border Industrial Complex and its conceptual variations.

This was stressed also in the conclusions of the already mentioned EU Parliament Review of security measures in the 7 ^th^ Research Framework Programme FP7 2007–2013, stating that: « *The policymaking process on security research sidesteps a number of societal actors. This is reflected both in the high-level Public-Partner Dialogue and in the second-track expert groups tasked with defining security research, where representatives of security industry and public security bodies are overwhelmingly present, at the expense of actors who may speak in the name of the citizens, including MEPs or non-governmental organisations. The unequal representation of industry, security agency and civil society in the policymaking process helps interests of the latter*» ( Bigo *et al.*, 2014, 33). The analysis of the Commission's suggestions for an EU security industrial policy further demonstrates further that « *the question of fundamental freedoms and rights is reduced to a matter of commercial considerations and as a limit to the acquisition of otherwise high-performance products. We can thus anticipate that funded security research in the future will be mainly put at the service of industry rather than society*» ( Bigo *et al.*, 2014, 7).

Furthermore, Statewatch and TNI examined from the same angle the case of the European Organisation for Security (EOS), the main lobby group for the security sector in Europe. Through the three year, FP7-funded ARCHIMEDES project (EU contribution: €1.4 million), EOS proposed the adoption of a tailored model to promote a “common innovation culture” and align research agendas between EU and member states: the « *end-to-end approach for security research and innovation*» that proposes the formal integration of public authorities and private companies in a new type of “governance” structure. This was to be achieved by bringing together so-called end-users and operators into a « *permanent public-private dialogue*» in order to reinforce cooperation with the supply side, that is, the industry. The broadest range of actors participating in this debate, according to EOS's final report, would represent the best course of action. However, several critics pointed out that the platform as it is presented by the report « *would be made up of pretty much anyone with an interest in security, except for citizens and their elected representatives*» ( Jones, 2017, 34). EOS itself has praised a democratic approach that involves the broadest possible range of people, but 12mportan stated that such an approach needs to be tempered, as the dialogue had to be taken « *in a closed and trusted environment that allows (when needed) sharing of confidential information*», such as those already been established as part of Archimedes through a number of industry stakeholder roundtables organised and led by EOS itself ( Jones, 2017, 35). Archimedes final report states that concerns over fundamental rights are « *politically correct but not necessarily a competitive advantage at MS and international level*» ( Jones, 2017, 36).

Other scholars analyse these dynamics focusing on Frontex. In their report *Lobbying Fortress Europe. The making of a border-industrial complex* the authors frame the increasing relationships between the EU border agency and “arms, surveillance, and biometrics companies” as a « *significant overlap between the companies that directly lobby Frontex and the companies that benefit from EU procurement for building Europe’s walls, both physical and virtual*» ( Douo *et al.*, 2021). In this perspective, in its *Decision of 18 April 2018 on discharge in respect of the implementation of the budget of the European Border and Coast Guard Agency (Frontex) for the financial year 2016 (2017/2164(DEC)*, the European Parliament, indeed, demanded Frontex to introduce a system of lobby transparency, consisting in a transparency register and in the disclosure of all its meeting with third party stakeholders.

In a recent report, a prominent group of scholars argue that « *forging a tight relationship between Frontex, industry, and member states therefore delivers a win-win-win scenario for these three parties*» but « *in absence of sufficient democratic control and oversight, people’s rights – in particular, the fundamental rights of people who migrate – are not only disregarded, but perpetually endangered*». In particular, the authors insist that « *groups working to defend human rights are left on the side-lines*» of a border-industrial complex in the making ( Douo *et al.*, 2021). The authors emphasize that none of the 138 private organizations that Frontex met with between 2017 and 2019 were human rights organizations, and that only one of them was an NGO. After analysing the documentation produced following 17 industry meetings held during the same timeframe, the report also notes that « *omission from almost every one of these discussions is the potential impact on human rights of these technologies and products, including undermining people’s fundamental right to privacy, presumption of innocence, and liberty*» ( Douo *et al.*, 2021).

The authors analysed industry presentations and concluded that in these documents migrants were considered not « *as subjects of border crossing but rather objects that need to be managed*» ( Douo *et al.*, 2021), presenting it more like the target of an industrial process rather than stressing the human dimension of this “target”, operating a sort of complete dehumanization of the target of the product, not considering at all the fundamental rights dimension, or considering 13mpor merely secondary.

Ruiz has a similar viewpoint; after analysing Frontex’s founding regulations and mandate, the author claims that the « *EU considers migration a security risk on a par with crimes such as drug trafficking or smuggling, paving the way for the approval of exceptional measures to address it*» ( Ruiz Benedicto, 2019, 6). Furthermore, considering 19 main joint operations carried out by Frontex (2005–2018) to analyse the “practice of securitisation” implemented by the Agency, Ruiz comes to the conclusion that none of these joint operations conducted had a specific mandate to rescue people or to include civilian shipping fleets in its actions, focusing almost exclusively on fighting different cross-border crimes, most of which related to flows of migrants; thus insisting on security of borders rather than on security and well-being of migrants ^24^. In other words, migrations are treated by Frontex more as a criminal matter rather than a humanitarian issue that poses relevant problems when it comes to the respect of human rights of migrants. Ruiz clearly states that in the framework of these operations, Frontex adopts security practices that criminalise people who are fleeing from poverty, war and violence. It also plays an active role in operations to return migrants to their country of origin. Accordingly, humanitarian and civilian organisations are actively prevented to rescue people in distress ( Ruiz Benedicto, 2019, 6–19).

### Frontex and the marginalization of HRGs and CSOs: the case of the Consultative Forum

As said, from its creation Frontex has been criticised for its tendency to disregard human rights in the implementation of its activities. Due to this criticism, EU institutions have emphasized Frontex's dedication to promoting and upholding “behavioural standards" in a way that reflects the principles of the Charter of Fundamental Rights. So, the Regulation (EU) No 1168/2011 established the Consultative Forum on Fundamental Rights to ensure that fundamental rights are being upheld.

The Consultative Forum began operating in 2012, and included nine civil society organizations, four international organisations – i.e., OSCE/ODIHR, Council of Europe, UNHCR, IOM – and two other EU agencies, namely the European Asylum Support Office (EASO) and the Agency for Fundamental Rights (FRA). The Consultative Forum was downsized to fourteen people in January 2020. Several pertinent organizations, including the AIRE center, Caritas Europa, ECRE, and PICUM, are no longer a part of the Consultative Forum in this most recent composition. In accordance with Article 108 of the Regulation No 168/2011, the role of the Consultative Forum is to assist the Agency « *by providing independent advice in fundamental rights matters*». To this end, any issue pertaining to fundamental rights may be discussed within the Consultative Forum. The Consultative Forum creates annual work plans in which it lays out the goals and tasks to be accomplished in order to better guarantee that Frontex activities are aware of essential aspects of fundamental rights and to offer strategies for more effectively promoting their respect in Agency operations. Annual reports from the Consultative Forum give a summary of the activities the organization has been involved in.

In principle, the Consultative Forum offers a unique chance for CSOs and other organizations to influence Frontex from within, strengthening the Agency’s commitment to fundamental human rights. Members of the Consultative Forum have the option of requesting information and documents directly from Frontex regarding basic rights-related problems pertaining to the Agency’s operations. Additionally, members have begun actively participating in Frontex operations since 2014, including the highly contested return operations, as well as other operations at land and sea borders.

However, according to the key findings of the Final Report *External Evaluation of the effectiveness and impact of Frontex Consultative Forum on Fundamental Rights* published in July 2019 as well as from the Consultative Forum yearly reports of 2017, 2018 and 2019 (the last publicly available), several factors hamper the Consultative Forum activity, such as: the lack of consultation and implementation of the Consultative Forum’s advice, the problems faced by CSOs and HRGs in getting access to relevant information, and the understaffing and availability of Consultative Forum’s members.

As for the first issue, the lack of consultation and implementation of the forum’s advice, Douo, Izuzquiza and Silva, pointed out that the Forum may be consulted on any matter pertaining to basic rights, although Frontex ultimately decides when and which issues to consult on. For instance, as evidenced by the work programmes of the Consultative Forum, none of the issues that the Agency discussed with industries in the meetings examined in the report or, more generally, « *in relation to procurement of technologies or relationships with security companies*», were subject to consultation ( Douo *et al.*, 2021).

The 2019 report discusses the implementation of the European Integrated Border Management Strategy (EIBMS), highlighting its importance, since it is likely to provide guidance in the process and influence the overall Fundamental Rights Strategy of the Agency. In this perspective, the Consultative Forum expressed concern that « *regrettably, and despite initial discussions, the expertise and advice of the Consultative Forum were not sought in the development of the Frontex European Integrated Border Management Strategy*» (p. 23). Other instances where the Consultative Forum's counsel was not requested are included in the 2019 report, despite the fact that the topics in question would fall inside the Consultative Forum's expertise and mandate. For instance, in contrast to previous years’ practices and in spite of Frontex’s expanding duties in this area, the Consultative Forum was not consulted for guidance on the creation and execution of Frontex training programs and courses in 2019 (p. 33).

The Consultative Forum’s 2019 report asserts also that Frontex blatantly disregarded its recommendations in at least other two cases, which are connected to the broader framework of European external border control regulations. Concerns have been voiced, for instance, on the potential impact of enhanced Frontex aircraft surveillance over the Central Mediterranean and the disclosure of information to the Libyan Maritime Rescue Coordination Centre about search and rescue incidents that Frontex identifies. The Consultative Forum stressed the increased number of individuals being brought back « *to face arbitrary detention and horrific conditions in Libya*». Similar concerns were raised regarding Frontex’s Multipurpose Aerial Surveillance (MAS) in Croatia, whereas consistent reports of police violence and illegal pushbacks of migrants by Croatian authorities, as documented by media and various organisations, including those represented in the Consultative Forum, became public and available (p. 24).

The Consultative Forum’s suggestions regarding Frontex operational activities in Hungary are yet another situation that stands out. Highlighting the fact that Hungary was subject to a Commission infringement proceeding for its asylum laws and policies, the Consultative Forum suggested « *that Frontex refrain from supporting returns from Hungary and suspend any return-related activities from the country*», also given « *the direct responsibility of the Agency to ensure respect for the principle of non-refoulement in all its activities*». Frontex continued to provide operational support to Hungary in spite of this. This decision was driven by the idea that the Agency’s presence may help to improve the situation; as a result, Frontex even increased the number of employees stationed at the Serbian-Hungarian border. The Consultative Forum observes, however, that from its perspective, there was no real improvement of the overall situation (p. 25).

Another important issue highlighted in the external evaluation is that of consultation and implementation of the Forum’s advice. In this perspective, the evaluation established that, despite the fact the Forum’s work was described as relevant and useful by Frontex staff, « *an important number of the Forum’s recommendations and opinions did not translate into concrete actions or were only partially incorporated by the Agency*» (p. 114), although recommendations are usually operational and targeted. The external evaluation stresses also that: « *The Forum’s recommendations and opinions are not binding and merely advisory, and that Frontex has discretionary competence to decide whether (and how) to follow the Forum’s advice on a specific issue*». Frontex had no obligation « *to reply the Forum’s recommendations/opinions or to inform the Forum on any actions taken on the basis of their advice*» (p. 115). However, it 15mporta noted that failure to implement them hampered a better protection of fundamental rights in Frontex’s activity. The external evaluation concludes that the Agency will be required to update the Consultative Forum on the follow-up on its suggestions under the new Frontex Regulation that was proposed in September 2018, which is meant to increase transparency on this matter (pp. 93–94).

The second factor hampering the Consultative Forum activity and effectiveness is the issue of access to information. As pointed out by Giannetto, in the last years the Consultative Forum « *has repeatedly lamented issues with access to information, which should be effective as per art. 108 of the EBCG regulation (2019/1896)*» ( Giannetto, 2020). A review of the Consultative Forum’s yearly reports confirms this analysis ^25^.

The issue of accessibility and the provision of pertinent information to the Consultative Forum are both considered by the external evaluation too. In this regard, the evaluation report stresses that, while interviewing Consultative Forum’s members, « *the need to access information about the respect of fundamental rights*» have been identified as « *one of the main challenges faced by the Forum in fulfilling its mandate*» (p. 133). Although the Consultative Forum standardized its processes for asking Frontex for information on matters pertaining to fundamental rights, it seems that Frontex still takes time to respond to these requests, and in several instances the information provided, particularly operational plans, were incomplete, as was the case with operational activities carried out by the Agency in Hungary (2019, p. 134). Furthermore, it appears that requesting information from the Agency has become a very time-consuming activity for Consultative Forum’s members.

The third factor which significantly hampers the Consultative Forum activity, according to the external evaluation, is understaffing and availability of Consultative Forum’s members. The Consultative Forum’s sixth annual report for 2018 claims that Frontex’s capacity to uphold its obligations in the field of fundamental rights was inhibited by the understaffing of the Agency’s Fundamental Rights Office. Since 2014, this has been a constant problem: adequate staffing is paramount to ensuring the implementation of fundamental rights obligations under the EBCG regulation and is presented as particularly relevant due to the future expansion on Frontex’s mandate. In this persspective, the Consultative Forum’s claims that the Fundamental Rights Office’s work is being compromised in important areas due to the understaffing situation, such as « *monitoring of operations, handling of complaints, provision of advice on training, risk analysis, third country cooperation and return activities*» (p. 19).

Most of the stakeholders who participated in the evaluation process highlighted a significant lack of human resources, both in terms of the staff deployed and the constrained time that Forum members have to contribute to the work of the Forum, according to the evaluation report included in the seventh annual report of the Consultative Forum of 2019. These factors would affect the Consultative Forum’s ability to carry out its mission. On the one hand, most of the stakeholder suggested the implementation of « *an independent and permanent Secretariat with sufficient budget and human resources to effectively assist the Forum not only the administrative tasks but also with the substantive work*» (p. 21). On the other hand, consulted stakeholders expressed concern that some CSOs find it difficult to keep up with the work of the Consultative Forum and continue with their work in their respective organizations at the same time because membership in the Consultative Forum does not provide for compensation. It was suggested as a solution to this issue to provide some form of monetary compensation for the efforts made by Forum participants. (pp. 145–146).

### The deployment of SOSTs in border surveillance and the role/perception of HRGs and CSOs

This section analyses the specific issue of the SOSTs deployed in the framework of border controls. This issue is closely connected to both the involvement of HRGs and CSOs in decisional processes related borders’ management and surveillance and the attention given by EU institutions to the human rights’ dimension. By this point of view there is a specific focus on the relationship existing between CSOs, HRGs and European Institutions in drafting and approval process of two EU regulation instruments: the Smart Borders Package (SBP) and the legislative proposal on Artificial Intelligence (AI).

Starting with the consultations held between EU institutions and HRGs/CSOs on the SBP regulation, related to the use of biometrics and the duration of retention of personal data collected during border controls, the 2013 version of the Smart Borders Package – then approved in an amended version in 2017 – consisted of three proposals: first, the creation of an Entry/Exit System (EES), aimed at replacing manually procedures on third-countries nationals’ passports with a digitalised stamping system and automatic overstays calculations; second a Registered Traveller Programme, providing a pre-screening option for frequent third country travellers, so to access EU territory through automated border check systems following Member States’ nationals standard procedures; and third an amendment of the Schengen Borders Code to update existing regulations with the technical changes needed to implement the abovementioned systems.

The purpose of the consultation was to obtain « *views and opinions to underpin the ongoing impact assessment of the Smart Borders Package (...) and gather new ideas and knowledge*» and it was organised around a set of questions as follows: use of biometric identifiers, process to accelerate border crossing for non-EU citizens, data, law enforcement access to the Entry/Exit System data, stamping, comments/other questions (impacts on asylum seekers, impacts on those who may need to issue invitations, other comments). For the purpose of this analysis, the sets on “use of biometric identifiers” and “data” have been taken into consideration, as well as the question on the impact on asylum seekers, as migration policies are often under the lens of many civil society organisations. Among the 39 contributions available at the Consultation’s dedicated Web page, 9 of them were submitted by civil society organisations, namely: 5 NGOs, 2 confessional organisations. 1 University, 1 transportation workers’ union. There were two scales of evaluation which were implicitly proposed by the survey itself: the fairness and the efficacy of the proposed measures.

The positions of each CSOs are not included here for obvious reasons of spac 17mport it is possible to summarise affirming that the results of the questionnaires were fully in line with what proposed by Pavone and Degli Esposti (2010) when the two authors affirmed that a trade between security – i.e., the efficacy of proposed technologies – and privacy – i.e., the fairness of proposed technologies – does not seem to take place. Instead, organisations which contest the efficacy of the use of biometrics link their concerns with potential infringements of fundamental rights such as privacy, family life, freedom of movement etc. On the other hand, there are organisations which affirm that the efficacy of those technologies do not foresee issues related with the fairness of proposed measures. In their research, Pavone and Degli Esposti find out that some of the main concerns expressed by the participants were first related to potenti mportanti s sesses (being migrations a very sensitive political matter) and, more generally, an infringement of the democratic framework; second, to uncertain professional and moral profile of surveillance operators, an issue which calls for clear rules and reliable mechanisms of sanction. It seems that there is no trade off at all between the two opposite dimensions of security and privacy. How to tackle this issue?

The two authors also indicate that the context – that is, the reason why a particular technology was chosen to be deployed – rather than the technology itself appears to be at the heart of participants’ worries. Naturally, in such a scenario, dependability on the institutions in charge of the deployment of chosen technology is inherent. Because trust cannot be viewed as a good to be exchanged, it should be created as a result of citizens’ involvement (the perspective adopted when considering privacy and security as a trade-off relationship). The proposed framework does, in fact, echo the stance taken by HRGs during the Consultation on the Smart Borders Package as previously described ^26^.

Continuing with the analysis of the legislative proposal on Artificial Intelligence (AI) to be used in borders’ controls, in January 2021 a large group of HRGs and CSOs, including those gathered under the European Digital Rights (EDRi) umbrella association, wrote an open letter to the EU Commission, calling for the introduction of “red lines”, i.e., regulatory limitations in order to guarantee fundamental rights vis-à-vis the implementation of artificial intelligence (AI) tools (i.e. drones and biometric systems) in border surveillance. The signatory organisations focused their attention, among other topics, on biometric mass surveillance, and on the use of AI at the border and in migration control. According to their letter, « *AI has been tested to purportedly detect lies for the purposes of immigration applications at European borders and to monitor deception in English language tests through voice analysis, all of which lack credible scientific basis. In addition, EU migration policies are increasingly underpinned by AI systems, such as facial recognition, algorithmic profiling and prediction tools for use within migration management processes, including for forced deportation*» (p. 2). Stating that such uses may infringe on data protection rights, the right to privacy, to non-discrimination and to seek asylum, the signatories propose a ban or a moratorium on the use of AI at borders, until their compliance with international human rights standards will be assessed by an independent part.

Also in this case, the position expressed by the organisations signing the letter overlaps the two only apparently opposed and complementary issues: the real efficacy of the proposed AI developments; and the fairness of AI indiscriminate application in the context of borders control. But what is even mo mportanti s is tha the letter was not considered by the EU in the process of adoption of the regulation, which regularly entered into force not considering these concerns and questions.

## Conclusions

This non-systematic review aimed at exploring the perceived marginalisation that HRGs and CSOs claim and denounce in relation to border management and surveillance at European level.

First, the text presented a press review on EU relevant public debates involving the issues of border control and human rights showing that, in mainstream media, criticism on SOST and their use in this framework remain on the background. Instead, policies and practices that make use of such technology to achieve border management goals appear to be the main issue at stake. Second, Frontex’s legal framework, guidelines and Code of Conduct were analysed to pinpoint the increasing weight of the concept of fundamental rights protection in the legal basis of the Agency’s action and mandate. Third, the review focused on the concept of “border-industrial complex” as one of the main theoretical frameworks used by HRGs and CSOs to criticize the increasing political and financial influence of industrial security lobbies in border management, at the expense of a more human rights-oriented approach. Then, to present a case study on this perceived marginalisation, the case of Frontex’s Consultative Forum on Human Rights was outlined focusing on the main factors that, according to the evaluation reports, hinder the effective functioning of this consultative body. Finally, the review focused on how participatory tools used by EU institutions to engage HRGs and CSOs on migration policies and border management/surveillance have been received and implemented.

It is quite difficult to compose an overall mapping of every critical position expressed by human rights groups on surveillance technologies and border control. A huge number of statements, inquires, complaints, allegations and claims have been produced in the last years. As they often share a common ethical ground, these stances differ one from the other according to the specific field of action of every group – being it territorially or thematically defined. Such a differentiation guarantees, in turn, a specific setting of action and spaces of self-organisation and self-representation for any of the abovementioned HRGs. The attempt of this analysis is thus to focus on a relevant aspect which seem to be shared by many of these groups: the perception of a progressive disregard of issues and priorities related to human rights when defining border control policies and the deployment of surveillance technologies; HRGs consider indeed themselves as marginalised actors in the framework of border control policy-making arena.

In the framework of ARESIBO, this non-systematic review had the purpose to provide insights to elaborate a participatory model that, addressing the perceived marginalisation of HRGs and CSOs, could foster their engagement is SOST design. The ARESIBO Participatory Model (APM) is a rather innovative participatory model built on the previous experiences and practical knowledge acquired in significant projects by one of the ARESIBO consortium’s partners (i.e. ISIG – Institute of International Sociology), and it represents a working proposal and a practical tool to tackle the perceived marginalisation of HRGs and CSOs. The APM has been developed and tested in the framework of ARESIBO activities and it has been elaborated upon the participatory techniques developed and implemented by ISIG in the framework of its cooperation and activities with the Council of Europe, as well as on the theoretical model on participatory tools proposed by Ostrom and Gardner in 1994 (i.e., the Institutional framework of Common-pool resources management – Ostrom, Gardner and Walker, 1994, and the Civil participation framework of the Council of Europe – ISIG/CoE 2017).

The detailed description of the functioning and of the structure of the APM will be included in the next paper of the research diptych based on the results of the ARESIBO project named: *From marginalization to engagement of citizens in SOSTs decision-making: participatory models setting a common ground for border surveillance and respect of fundamental rights. The case of ARESIBO H2020 project*.

The APM represents a working proposal that uses innovative tools and participatory techniques to tackle the perceived marginalisation of HRGs and CSOs, as well as a model that can be applied – after further appropriate testing and dissemination – in similar contexts and situations.

## Data Availability

All data underlying the results are available as part of the article and no additional source data are required.

## Footnotes

^1^ « *Frontex, the European Border and Coast Guard Agency, promotes, coordinates and develops European border management in line with the EU fundamental rights charter and the concept of Integrated Border Management*». Please see: https://frontex.europa.eu/about-frontex/who-we-are/origin-tasks/.

^2^ The APM is thoroughly presented in the methodology article “Towards the engagement of citizens in SOSTs decision-making: participatory models setting a common ground for border surveillance and respect of fundamental rights. The case of ARESIBO H2020 project”, also published on the Open Research Europe platform.

^3^ « *The principle of non-refoulement forms an essential protection under international human rights, refugee, humanitarian and customary law. It prohibits States from transferring or removing individuals from their jurisdiction or effective control when there are substantial grounds for believing that the person would be at risk of irreparable harm upon return, including persecution, torture, ill treatment or other serious human rights violations*». This principle is included in all major conventions and international instruments dealing with the protection of human rights in general (e.g., e Convention against Torture and Other Cruel, Inhuman or Degrading Treatment or Punishment and the International Convention for the Protection of All Persons from Enforced Disappearance), as well as at a regional and European level (e.g., art. 18, Right to Asylum Charter of Fundamental Rights of the European Union). However, probably the most important documents which developed and applied the non-refoulement principle consistently and continuously have been the 1951 UN Refugee Convention and the 1967 Additional Protocol, both dealing with refugees and asylum seekers and confirming the centrality of this legal principle. Please see:
https://www.ohchr.org/sites/default/files/Documents/Issues/Migration/GlobalCompactMigration/ThePrincipleNon-RefoulementUnderInternationalHumanRightsLaw.pdf for more details.

^4^ Frontex has been established in 2004 and is the EU Agency created for the coordination of external borders management by EU countries and Schengen associated countries. It also helps to harmonise border controls across the EU. The agency facilitates cooperation between border authorities in each EU country, providing technical support and expertise. As stated on the Agency website Frontex is the Agency in charge of « *ensur[ing] safe and well-functioning external borders providing security*». https://frontex.europa.eu.

^5^ euro|topics. https://eurotopics.net/.

^6^ The complete list of the newspaper articles resulting from the research has not been included in this paper for obvious reasons of space.

^7^ The analysis proposed by ARESIBO presents also four other relevant dimensions which are somehow related to human rights and border management, even if they are not directly related to the approach and the field of analysis of this paper. It is important nevertheless to briefly recall them here. The first one is the role those third-party countries, particularly Turkey and Libya, play in the so-called “outsourcing border control” debate which is highlighted in 18 out of 51 articles that address the possibility of moving forward, re-framing, or giving up with such a strategy. They address the reality that the EU relies on external nations to control migratory flows – countries that often do not recognize the most significant human rights conventions. The second is that 7 articles out of 51 stress the issue of solidarity among EU member States in managing the burden of migration flows, which according to some of the selected sources lies mostly on the most exposed countries like Italy, Spain, Malta and Greece. Thirdly, 4 out of 51 articles agree that the existing structure of EU migration laws actually encourages illegal immigration, creating the circumstances for the market for human trafficking and people smuggling to thrive. Finally, only 4 articles out of 51 explicitly quote Frontex.

^8^ The 1951 Refugee Convention and its 1967 Protocol are considered as the key legal documents that form the basis of the work of the UN High Committee for Refugees (UNHCR): « *With 149 State parties to either or both, they define the term ‘refugee’ and outlines the rights of refugees, as well as the legal obligations of States to protect them. The core principle is non-refoulement, which asserts that refugees should not be returned to a country where they face serious threats to their life or freedom. This is now considered a rule of customary international law. UNHCR serves as the ‘guardian’ of the 1951 Convention and its 1967 Protocol. According to the legislation, States are expected to cooperate with [UNHCR] in ensuring that the rights of refugees are respected and protected*». See https://www.unhcr.org/1951-refugee-convention.html.

^9^ See https://www.echr.coe.int/documents/convention_eng.pdf.

^10^ REGULATION (EU) No 1168/2011 OF THE EUROPEAN PARLIAMENT AND OF THE COUNCIL of 25 October 2011 amending Council Regulation (EC) No 2007/2004 establishing a European Agency for the Management of Operational Cooperation at the External Borders of the Member States of the European Union. REGULATION (EU) No 1168/2011 OF THE EUROPEAN PARLIAMENT AND OF THE COUNCIL of 25 October 2011 amending Council Regulation (EC) No 2007/2004 establishing a European Agency for the Management of Operational Cooperation at the External Borders of the Member States of the European Union. REGULATION (EU) 2016/1624 OF THE EUROPEAN PARLIAMENT AND OF THE COUNCIL of 14 September 2016 on the European Border and Coast Guard and amending Regulation (EU) 2016/399 of the European Parliament and of the Council and repealing Regulation (EC) No 863/2007 of the European Parliament and of the Council, Council Regulation (EC) No 2007/2004 and Council Decision 2005/267/EC. REGULATION (EU) 2019/1896 OF THE EUROPEAN PARLIAMENT AND OF THE COUNCIL of 13 November 2019 on the European Border and Coast Guard and repealing Regulations (EU) No 1052/2013 and (EU) 2016/1624.

^11^ It is important to recall briefly here the most important articles of the Regulation No. 1168/2011 referring directly to fundamental rights: art. 9 (rights of refugees and asylum seekers), art. 18 (inclusion of fundamental rights in the training of Frontex’s personnel), art. 22 (fundamental rights and relations with third countries), art. 29 (importance of human rights in relation to Frontex’s overall mandate). Relevant is also art. 26a which includes substantial provisions related to the obligations of Frontex towards the respect of fundamental rights: a) art. 26a 1 claim for drawing up and further developing a Fundamental Rights Strategy and for « *an effective mechanism to monitor the respect for fundamental rights in all the activities*»; b) art. 26a 2 calls for establishing a Consultative Forum for Fundamental Rights « *to assist the Executive Director and the Management Board in fundamental rights matters*»; c) art. 26a 3 provides for designing a Fundamental Rights Officer « *independent in the performance of his/her duties*» and reporting « *directly to the Management Board and the Consultative Forum. (…) contribut(ing) to the mechanism for monitoring fundamental rights*»; d) art. 26a 4 states that the Consultative Forum and the Fundamental Rights Officer shall have « *access to all information concerning respect for fundamental rights, in relation to all the activities of the Agency*».

^12^ Regulation No 2016/1624 Art. 2: « *The aim is to manage the crossing of the external borders efficiently and address migratory challenges and potential future threats at those borders, thereby contributing to addressing serious crime with a cross-border dimension and ensuring a high level of internal security within the Union. At the same time, it is necessary to act in full respect for fundamental rights and in a manner that safeguards the free movement of persons within the Union*».

^13^ Regulation No 2016/1624 Art. 14: « *The extended tasks and competence of the Agency should be balanced with strengthened fundamental rights safeguards and increased accountability*».

^14^ Regulation No 2016/1624 Art. 47: « *The European Border and Coast Guard, which includes the Agency and the national authorities of Member States which are responsible for border management, including coast guards to the extent that they carry out border control tasks, should fulfil its tasks in full respect for fundamental rights, in particular the Charter of Fundamental Rights of the European Union (‘the Charter’), the European Convention for the Protection of Human Rights and Fundamental Freedoms, relevant international law, including the United Nations Convention on the Rights of the Child, the Convention on the Elimination of All Forms of Discrimination Against Women, the Convention Relating to the Status of Refugees and obligations related to access to international protection, in particular the principle of nonrefoulement, the United Nations Convention on the Law of the Sea, the International Convention for the Safety of Life at Sea, and the International Convention on Maritime Search and Rescue. In accordance with Union law and those instruments the Agency should assist Member States in conducting search and rescue operations in order to protect and save lives whenever and wherever so required*»

^15^ The most relevant and innovative articles of the Regulation No. 2019/1896 referring directly to fundamental rights are (the complete text of these articles will not be recalled here for obvious reasons of space): art. 1 (the role of fundamental rights in the Agency’s overall mandate); art. 24 (the role of fundamental rights in balancing the extension of tasks and competences of the Agency and the weight of fundamental rights in Frontex’s tasks and competencies); art. 42 (the respect of fundamental rights in the management of external borders); art. 55 (the respect of fundamental rights in hotspot areas); art. 78 (the inclusion of fundamental rights in the training of Frontex’s personnel); art. 81 (the role of fundamental rights in return decisions and operations); art. 91 (the role of fundamental rights in negotiations and collaborations with third countries); art. 104 (the establishment and implementation of a complaint mechanism to safeguard the respect for fundamental rights in all the activities of the Agency).

^16^ In addition to that the Strategy addresses the issue of personal information, stating also that « *Any processing of personal data, including gathering, retention, exchange and deletion of information, shall be handled with respect for fundamental rights, including the right to private and family life and protection of personal data*» (p.12). In regard to the collection of biometric data, the document highlights that this shall be done « *only when authorised by law and with respect for integrity of the person, with provision of sufficient and adequate information, refraining from the use of force, and in an age-, gender and culturally sensitive manner, respecting the EU and international legal framework*» (p.12). Finally, for what concerns automated tools and the use of artificial intelligence in border management, the document stresses that these tools « *shall always be used in full compliance with EU data protection legislation and with appropriate safeguards, and in line with human dignity and the principle of non- discrimination, and shall be monitored, including through personal oversight, and verified regularly*» (p.12).

^17^ « *Participants must: a) promote, respect, fulfil and protect the right of persons seeking international protection to be identified, receive adequate assistance and be informed in an appropriate way, preferably using a number of the most common languages that third-country nationals understand or are reasonably believed to understand, about their rights and relevant procedures*» (p. 12).

^18^ « *Participants, whether on or off duty, must: (…) b) act with fairness, impartiality and non-discrimination and treat all persons with dignity, courtesy and respect; (…) e) not be ignorant of cultural differences when interacting with persons so as to cause misunderstandings*» (p.14).

^19^ It seems useful to remind here that in addition to the Code of Conduct, the Agency created a separate *Code of Conduct for return operations and return interventions* that Frontex supervised or organized in collaboration with the Fundamental Rights Office and Consultative Forum. The purpose of this document, which serves as a supplement to the Code of Conduct's rules, is to precisely govern Return Operations (RO) and Return Interventions (RI) in full respect of the fundamental rights of the returnees.

^20^ The definition had risen to the honours of the story due to a well-known intervention of US President Dwight D. Eisenhower in 1961, when he warned the country about the huge influence which the “ *military-industrial complex*” was progressively acquiring in the country.

^21^ The political debate does not diffusely account for this framing as well, even though some exceptions can be found as, for instance, the *Review of security measures in the 7th Research Framework Programme FP7 2007–2013*, a study delivered in 2014 for the LIBE Committee – European Parliament Committee on Civil Liberties, Justice and Home Affairs ( Bigo *et al.*, 2014).

^22^ The External Borders Fund (2007–2013) amounted to €1.76 billion and its successor – the so-called Internal Security Fund Borders (2014–2020) – to €2.70 billion while the new Integrated Border Management Fund (IBMF) (2021–2027) will reach €12.49 billion ( Akkerman, 2020). Same figures are included also in the Communication from the Commission COM(2020) 442 final of the 27.05.2020: “The EU budget powering the recovery plan for Europe”.

^23^ The report provides also an analysis of the distribution of research funding by theme in the European Security Research Programme 2007–2013, supporting two main considerations: 1. The substantial matching of the structure and the topics with those proposed by the European Security Research Advisory Board (ESRAB) in the report *Meeting the challenge* published in 2006. ESRAB composition reflected the weight of industry in such a body: of the 50 seats, 14 were indeed occupied by defence and security industries, 18 by Member States, 14 by academics and research institutes, 2 to the European Defence Agency and Europol and finally 2 to groups described as “civil liberty groups and think tanks” ( Hayes, 2009, 15–18). 2. Projects were undertaken by consortiums primarily made up of corporations and other companies, research organisations, higher education institutes, state agencies and ministries. Corporations and major technical research institutes took the majority of funding, respectively corresponding to 40% (€552 million) and 25% of the total €1.4 billion budget ( Hayes, 2009, 15–18).

^24^ Only in 1 operation – *Vega Children*, aimed at controlling cross-border movements of child migrants –, there was an actual cooperation between Frontex and a humanitarian organisation, the United Nations High Commissioner for Refugees (UNHCR). Documents on joint operations were found on government websites but were not mentioned in the Frontex annual reports, indicating some irregularity in the transparency of these reports

^25^ In the *Fifth annual report of the Consultative Forum* of 2017, there is a paragraph entitled “Limitations in providing the Forum with access to information”, where it is stated that: « *the Forum continues to face serious and further limitations particularly in relation to relevant operational reference and guiding documents. Given the constraints these places on its work, the Forum repeatedly raised this concern with Frontex management. A final response or constructive proposal from Frontex in this regard was yet to be received by the end of 2017*» (p. 17). On a comparable note, in the 2019 report the Consultative Forum regrets that its delegation was not allowed to acquire first-hand information by directly observing Frontex officers in Albania while in the performance of duties such as screening, debriefing and border surveillance. Following the document, this possibility would have constituted a crucial step towards understanding the role of the Agency in third countries and its Joint Operation in Albania (p. 33).

^26^ However, it's interesting to note that not all of the issues raised during the consultation process are viewed as valid concerns by all of the NGOs providing feedback. Indeed, Access Now and EDRi argued that the length of the data retention period should follow the ruling of the European Court of Justice rather than being discussed through a democratic process. This particular viewpoint clarifies the evidence that spaces of participation are criticized not only when they are perceived to be "too tight" to address demands and claims developed by NGOs, but also when they are perceived to be "too broad," thus raising questions about issues that must be taken into account as preconditions rather than negotiable positions.
