# Supplementary material for: The missing piece. The perceived marginalization of CSOs and HRGs in SOSTs’ decision-making processes: a review
Source: Open Res Eur. 2023 Jun 27;3:21. Originally published 2023 Jan 30. [Version 2] doi: 10.12688/openreseurope.15078.2 (PMC10643877; doi:10.12688/openreseurope.15078.2)
Supplement: Peer review history

# Reviewer report: Reviewer response for version 2

**DOI:** 10.21956/openreseurope.17146.r35746

**Published:** 2023-11-21

**Authors:** Sarah Perret

**Affiliations:** 1 ESPOL, Lille Catholic University, Lille, Hauts-de-France, France

The article is an output of the EU H2020 R&D project entitled “ARESIBO: Enhancing border security with augmented reality and enriched situation awareness”. The paper aims to review the perceived marginalisation of CSOs and HRGs, its reasons, and the elements that seem to make this marginalisation less substantial. Through a review of FRONTEX’s legal framework on human rights, and secondary sources such as press articles (mainly from *Euro/topics*), reports/documents (from NGOs, CSOs and FRONTEX), the article analyses why CSOs and HRGs feel marginalised from EU border security decisions and designs, and how their claims for the respect of human rights at the border are consider within the EU border security Agency, FRONTEX.

**General Comments**

 Overall, the article addresses a very interesting and important topic that is at the forefront of the controversies within border security field. The paper well presents the main issues shaping this question of the CSOs and HRGs participations in defining the EU border security and addressing the concern of their perceived marginalization. The paper is well structured and presents clear research questions about the perception of potentially marginalisation of HRGs and CSOs and the role played by the different stakeholders; the identification of the key views regarding the space of interactions between EU institutions and HRGs and CSOs; and finally the characteristics of the dialectic that is at stake between the issue of human rights and the one of EU border controls.

 However, several weaknesses remain. Indeed, some of the elements put forward need to be made more explicit, and above all the literature needs to be updated.

**Recommendations**

- The main weakness is the bibliography which ignores the many studies carried out about the actors and knowledge that make up security at the EU's borders, such as the work of Bigo, Jeandesboz, Bellanova and Duez, Leese, Martins and Jumbert, or Calcara, Csernatoni, and Lavallée. These studies bring other important elements to understand the dynamic that is at stake in the process of knowledge production on border security. But, above all, I believe that this paper also makes an important contribution to the debate on the place of critical actors and voices within the field of border security (Austin, Bellanova, and Kaufmann; Leese, Lidén, and Nikolova ; Martin-Mazé). Yet, this debate is not mentioned in the article.
- On several occasions, the authors provide no sources to explain or illustrate what they are referring to when they speak of “many HRGs and CSOs have reported and criticized several human rights violations in recent years” (p. 4) at the EU's borders. I suggest having a look on the several EU Ombudsman Decisions regarding FRONTEX activities (cf. 2014 Decision). It would be useful to provide 2 or 3 examples here.
- As already highlighted by other reviewers, the acronym SOSTs needs to be introduced and explained from its first use in the article to enable readers unfamiliar with the subject to understand what it is. It also seems important to detail what these SOSTs actually and materially refer to.
- The abstract should be updated to reflect the changes made between V1 and V2, in particular when it is mentioned that this is a 'systematic review', and the paper specifies that, in the end, it is a 'non-systematic review'.
- p. 8: It would be helpful for the reader to have at least one sentence to conclude/transition the section on Frontex’s legal framework on human rights, and another to explain why it is interesting to address the question of the links between the EU agency and the “military-industrial complex”.

Are all factual statements correct and adequately supported by citations?

Partly

Is the review written in accessible language?

Yes

Are the conclusions drawn appropriate in the context of the current research literature?

Partly

Is the topic of the review discussed comprehensively in the context of the current literature?

Partly

Reviewer Expertise:

Border, EU R&D programs, Migration, Political science, Security studies, Technology

I confirm that I have read this submission and believe that I have an appropriate level of expertise to confirm that it is of an acceptable scientific standard, however I have significant reservations, as outlined above.

# Reviewer report: Reviewer response for version 2

**DOI:** 10.21956/openreseurope.17146.r35754

**Published:** 2023-11-21

**Authors:** Clemens Binder

**Affiliations:** 1 University of Copenhagen, Copenhagen, Denmark

This paper aims to provide an overview over the perceived marginalization of Civil Society Organizations (CSOs) and Human-Rights Organisations (HROs) in decision-making processes about Surveillance-Oriented Security Technologies (SOSTs) in border security and migration control. The paper to this end draws on examples where these processes of perceived marginalization occur and provides empirical insights into relevant documents and structures of CSO involvement, such as the Frontex Consultative Forum on Fundamental Rights. It opens up a very crucial lacuna, which is the lack of civil society engagement with political processes in border management, specifically when it comes to technological aspects. Moreover, the paper aims to show that the missing CSO involvement is often a reason for problematic developments in the fields of human rights, as HROs are excluded. Moreover, the paper seeks to analyze modes of exclusion. While I command the ambition and research interest of this paper, it unfortunately suffers from serious shortcomings that undermine the quality of the analysis. It is a well-structured paper and the author’s prove ample knowledge of the topic itself. Nevertheless, in order to make this a good publication and valuable contribution, which it certainly has the potential to be, some larger improvements are needed.

- The paper lacks a clear thread that ties together the strands of analysis. By now, it seems as a scattered analysis of different relevant, interesting topics, but it is not made clear enough how these topics are connected in a larger context. Even with this being more of a review than an original research article, a good review requires thematical guidance. CSO and HRO involvement alone is very broad and therefore, the review might be systematic within the strands of analayis, but it lacks a clear connection between the different topics. It is stated that SOSTs are seen as a hook, however, hitherto that is not the case (see also below). However, I think a good way to resolve this issue could indeed be a stronger focus on SOSTs.
- The paper does not engage with a wide array of literature that is existent in the field, be it on Frontex’ negligence in terms of CSOs and human rights (Campesi 2022; Fjørtoft 2020; Glouftsios 2023; Perkowski 2021; Pollak and Slominski 2009) on the security industry and market (Binder 2020; Dadusc and Mudu 2020; Hoijtink 2014; Pacciardi and Berndtsson 2022) or on technologies (Bellanova and Glouftsios 2022; Glouftsios and Loukinas 2022; Leese 2022; Martins 2023; Pollozek 2020; Tazzioli 2018). That makes the claim of a systematic review somewhat weaker and undermines the quality of the analysis as such. However, also in terms of NGO reports, some of the reports are outdated and could be replaced by more current iterations.
- The paper, although claiming it being a central hook and mentioning it even in the title, does sideline the debate on SOSTs and pushes it to the end. That is a weakness that easily could be turned into a strength, as particularly in terms of connections to the security-industrial complex, there are important ties. SOSTs generally need to be integrated more into the analysis if they have a central role. Otherwise the paper promises something that it does not hold in its current version. Moreover, there is no clear definition of what a SOST is, including examples.
- I struggle to understand the role and significance of the media analysis in the paper. I’m not necessarily advocating for eliminating it, but in its current version, it seems detached and of little analytical value against the backdrop of the paper’s objectives. What do CSOs and HROs have to do with what emerges in a small sample of media articles? Here, either a clarification for the analytical position of this part is needed, or the part should be replaced by e.g. a more thorough analysis of SOSTs, which seem to be the main interest of this paper.
- There is only a superficial engagement with the security-industrial complex that is shaped very much by a few reports on this issue. The paper only mentions the Security Research Programmes very briefly, although they are fundamental parts of industrial involvement and shape the role of industry. For example, they do not look into the Protection and Security Advisory Group (PASAG) of Horizon 2020, which is a central forum for industrial involvement. Moreover, there needs to be a clearer problematization, i.e. – we already know that industry is strong and lobbies in technological programmes to a large extent, but what does that mean in the connection to CSOs and HROs? How is industry detrimental to human rights and CSO involvement. Also, what happens if CSOs interact with industry. Citing the reports is fine, but they also tend to scandalize a bit and put less focus on the very precise processes that come along with technology production, industry and security actors engaging.
- Similarly, the part on the Consultative forum could profit from a more concrete case study. The authors really have an opportunity there to delve into specific cases that were discussed by the consultative forum and highlight along the lines of one or two cases the critique and the argument they try to put forward.
- While it is an overview, sometimes the own contribution is missing. It is hinted that there is another paper, but without reading the other paper, this is more of a literature review that lacks a clear argument. That should not mean that the paper doesn’t have the chance to produce that, however for that purpose, there needs to be a clear improvement in terms of rationale and narrative. Also, the paper cannot expect the reader to have read the other paper, so the ARESIBO-material needs to be at least briefly introduced – not least because it is prominent in the introduction.

This is, all criticism aside, nevertheless an interesting and insightful read, which is also why I am advocating to eventually publish it. However, the points I have mentioned above leave me with some substantial reservations that need to be addressed before this paper goes out in the public. I am aware of this being a review, but also for a review, I suggest a clear argumentation, case studies in order to exemplify and a more integrated analysis. Nevertheless, I think the authors have done a good job in compiling this draft and I am confident they’ll be able to substantiate this article and deliver a valuable contribution to an important debate.

Are all factual statements correct and adequately supported by citations?

Partly

Is the review written in accessible language?

Yes

Are the conclusions drawn appropriate in the context of the current research literature?

Partly

Is the topic of the review discussed comprehensively in the context of the current literature?

Partly

Reviewer Expertise:

Border Studies, Migration Studies, Security Studies, European Union, Science and Technology

I confirm that I have read this submission and believe that I have an appropriate level of expertise to confirm that it is of an acceptable scientific standard, however I have significant reservations, as outlined above.

## References

[1] : Controlling the Schengen Information System (SIS II): The Infrastructural Politics of Fragility and Maintenance. *Geopolitics* .2022;27(1) : 10.1080/14650045.2020.1830765 160-184 10.1080/14650045.2020.1830765

[2] : Care without Control: The Humanitarian Industrial Complex and the Criminalisation of Solidarity. *Geopolitics* .2022;27(4) : 10.1080/14650045.2020.1749839 1205-1230 10.1080/14650045.2020.1749839

[3] : “Developing Future Borders. The Politics of Security Research and Emerging Technologies in Border Security.” In Emerging Security Technologies and EU Governance Actors, Practices and Processes, edited by Raluca Csernatoni, Antonio Calcara, and Chantal Lavallée. *Abingdon: Routledge* .2020;148-163

[4] : Policing Mobility Regimes: Frontex and the Production of the European Borderscape. Routledge Studies in Criminal Justice, Borders and Citizenship. *London New York: Routledge* .2022;

[5] : Performing secrecy: hiding and obfuscation in Frontex’s pushbacks scandal. *Journal of Ethnic and Migration Studies* .2023; 10.1080/1369183X.2023.2226825 1-21 10.1080/1369183X.2023.2226825

[6] : More power, more control: The legitimizing role of expertise in Frontex after the refugee crisis. *Regulation & Governance* .2022;16(2) : 10.1111/rego.12373 557-571 10.1111/rego.12373

[7] : Capitalizing on emergence: The ‘new’ civil security market in Europe. *Security Dialogue* .2014;45(5) : 10.1177/0967010614544312 458-475 10.1177/0967010614544312

[8] : Fixing State Vision: Interoperability, Biometrics, and Identity Management in the EU. *Geopolitics* .2022;27(1) : 10.1080/14650045.2020.1830764 113-133 10.1080/14650045.2020.1830764

[9] : EU border externalisation and security outsourcing: exploring the migration industry in Libya. *Journal of Ethnic and Migration Studies* .2022;48(17) : 10.1080/1369183X.2022.2061930 4010-4028 10.1080/1369183X.2022.2061930

[10] : Humanitarianism, Human Rights and Security: The Case of Frontex. InterventionsSource.2021;

[11] : Experimentalist but not Accountable Governance? The Role of Frontex in Managing the EU's External Borders. *West European Politics* .2009;32(5) : 10.1080/01402380903064754 904-924 10.1080/01402380903064754

[12] : Turbulences of speeding up data circulation. Frontex and its crooked temporalities of ‘real-time’ border control. *Mobilities* .2020;15(5) : 10.1080/17450101.2020.1801304 677-693 10.1080/17450101.2020.1801304

[13] : Security knowledges: circulation, control, and responsible research and innovation in EU border management. *Science as Culture* .2023;32(3) : 10.1080/09505431.2023.2222739 435-459 10.1080/09505431.2023.2222739

[14] : Spy, track and archive: The temporality of visibility in Eurosur and Jora. *Security Dialogue* .2018;49(4) : 10.1177/0967010618769812 272-288 10.1177/0967010618769812

# Reviewer report: Reviewer response for version 2

**DOI:** 10.21956/openreseurope.17146.r35751

**Published:** 2023-11-13

**Authors:** Denis Duez

**Affiliations:** 1 Institute for European Studies, Université Saint-Louis - Bruxelles, Bruxelles, Belgium

The paper reviewed is the first part of a research diptych carried out as part of a Horizon 2020 project called ARESIBO. The aim of the ARESIBO project is to improve the efficiency of border surveillance systems by providing the operational teams and tactical command and control level with accurate and comprehensive information related to border control.

 The paper explores the perceived marginalization of human rights groups (HRGs) and civil society organizations (CSOs) in border surveillance decision-making process at European level. It explains the main reasons for it, essentially from the point of view of HRGs and CSOs themselves. The text is well structured. The argument unfolds in three stages. The first stage aims at presenting a press review of EU relevant public debates involving the issues of border control and human rights in mainstream media, with a particular focus on the use of Surveillance-Oriented Security Technologies (SOSTs). The second stage examines the growing importance attached to the protection of human rights in the legal framework governing the activities of the Frontex Agency. Finally, the third stage consists of an analysis of the marginalization of HRGs and CSOs in the decision-making process, mainly through an analysis of the activities of the Consultative Forum on Fundamental Rights.

 The paper is well written in an accessible language. It deals with important and politically salient issues, highlighting the complex relations between protecting the fundamental rights of migrants and managing external borders of the European Union. From this point of view, the paper makes a relevant contribution to a highly topical debate.

 Despite its relevance, however, the paper has several weaknesses. Some of these weaknesses, were already present in the first version, and pointed out by one of the two reviewers.

- The meaning of the acronym SOST is still not explained in the paper's abstract or in the body of the paper. The full term "Surveillance-Oriented Security Technologies" is mentioned only once, in the list of keywords. Insofar as the expression used is not necessarily the most frequently used in academic work or in the most recent official documents to describe the use of new technologies in the field of border control, it would also be useful to clarify what types of technologies are covered by the concept of SOSTs. This clarification is all the more useful as the concept of SOSTs does not perfectly overlap with that of Smart Borders. Not all "smart technologies" are "surveillance technologies". For example, in the 2013 Smart Borders Package mentioned on pages 13-14, some smart technologies are indeed technological "border surveillance" devices (Eurosur), but others are more akin to technologies aiming at facilitating "border checks". On this point, we refer the authors to the terminological definitions in the Schengen Borders Code, which distinguishes between "border controls", "border checks" and "border surveillance" (art. 2).
- As with the concept of SOSTs, the scope of the concepts of Civil Society Organizations and Human Rights Groups is not clearly defined. Which organizations precisely fall within these concepts? Conceptual, terminological, and methodological choices that should have been explicitly justified are not explained. A more systematic presentation of the “pertinent organization” (p.12) could have been made.
- Although this criticism has already been formulated for the first version of the paper, the literature cited remains sparse, but also dated. Some of the works cited were published ten years ago. They have since been supplemented by more recent work (see Niovi Vavoula, Rocco Bellanova, Matthias Leese, Giorgios Glouftsios, or Didier Bigo and Julien Jeandesboz more recent works). Some specific concepts are also used without mentioning the authors who imported them in the field of migration-security studies or the debates surrounding them (for example, on the concept of "securitisation" discussed p. 9, see J. Huysmans, Th. Balzacq, etc.). Put briefly, the document does not really take account of the very rich (and recent) academic literature devoted to the question of the relationship between border controls, security technologies and human rights.
- The press review carried out using the euro-topics.net database is surprisingly limited. It is surprising that a keyword search covering a period of three years (2019-2020) on a subject as sensitive as migration and human rights results in a total of only 51 articles. One might have expected the choice of very broad keywords such as "border", "human rights" and "technology" to result in a much larger number of items. One can regret the lack of detail provided to the reader regarding the selection of keywords and the choice of the database. Eventually, the limited corpus raises not only the question of the systematic nature of the research, but also that of the validity of the conclusions drawn regarding the structuring of points of view around the issue studied.
- The text contains typos that should be corrected: p.4 "3mportanti", p.5 "5mportanti", p.6 "6mportanti", p.7, "7mportanti s7", etc.
- Finally, we have not read the paper constituting the second part of the research diptych, the one dealing with the participatory model. However, reading the title of this second part, "Towards the engagement of citizens in SOSTs decision-making: participatory models setting a common ground for border surveillance and respect of fundamental rights. The case of ARESIBO H2020 project", one wonder how the two texts fit together. The first deals with the participation of interest groups (CSOs and HRGs) in the European decision-making process, while the second deals with the participation of 'citizens'. These are two things that are certainly connected, but nonetheless distinct. This last comment is slightly outside the scope of this specific paper review, but the authors would probably benefit from arguing better about the connection between the two papers.

Are all factual statements correct and adequately supported by citations?

Partly

Is the review written in accessible language?

Yes

Are the conclusions drawn appropriate in the context of the current research literature?

Partly

Is the topic of the review discussed comprehensively in the context of the current literature?

Partly

Reviewer Expertise:

Border studies, security, irregular migration, Area of freedom security and justice

I confirm that I have read this submission and believe that I have an appropriate level of expertise to confirm that it is of an acceptable scientific standard, however I have significant reservations, as outlined above.

# Reviewer report: Reviewer response for version 1

**DOI:** 10.21956/openreseurope.16306.r30769

**Published:** 2023-02-20

**Authors:** Wojciech Opioła

**Affiliations:** 1 Department of Regional Studies, University of Opole, Opole, Poland

The reviewed paper: "The missing piece. The perceived marginalization of CSOs and HRGs in SOSTs’ decision-making processes: a review" is an engaging, non-systematic review of the literature and secondary sources concerning the issue of the role of CSOs and HRGs in border surveillance decision-making process.

 The review was done on some dimensions of current border surveillance discourse:

- review of media discourse
- analysis of legal and institutional framework (Frontex)
- analysis of the scientific literature about intersections between "security-industrial complex" and respecting fundamental rights in border surveillance,
- a case study of Frontex's Consultative Forum
- analysis of usage of participatory tools in border management and migration policies.

I have some minor critical remarks about the presented paper, however, my general impression is very positive. It is a comprehensive, detailed insight into a very up-to-date topic.

1. There is no explanation of the acronym SOST in the paper.
2. Authors stated, that the paper is a "systematic review", which I can't agree with. I only see elements of the systematic review method in a part about press debates. But even there, authors omit the most relevant criterion of systematic review: it must contain ALL relevant literature, and selection criteria must be described. It would be more proper if authors will use the term "non-systematic review", or "state-of-the-art analysis" instead. For systematic review methodology see for example PRISMA framework (Page, 2021)
3. Conclusions should be more comprehensive, and authors must state in this part, what is the general impression of analyses done in the paper? Now, the part conclusions are more the introduction to the second part of the paper, about the ARESIBO participatory model.
4. Cited literature should be enriched. I see only 17 references. If the aim of this paper is the review the state-of-the-art, more articles must be taken into consideration. For example, for the keyword "border surveillance", you can find more than 500 papers in the Scopus database.
5. Geographical scope of the analysis: paper is concentrated mainly on Mediterranean Region, with few references to the Hungarian-Serbian border. What with other EU borders (Poland/Belarus, Lithuania/Belarus, Finland/Russia, etc)? If these topics don't exist in the literature, isn't it interesting and worth an explanation, what is the reason behind that?

Are all factual statements correct and adequately supported by citations?

Partly

Is the review written in accessible language?

Yes

Are the conclusions drawn appropriate in the context of the current research literature?

Partly

Is the topic of the review discussed comprehensively in the context of the current literature?

Partly

Reviewer Expertise:

Cross-border cooperation, political science, border studies

I confirm that I have read this submission and believe that I have an appropriate level of expertise to confirm that it is of an acceptable scientific standard, however I have significant reservations, as outlined above.

## References

[1] : The PRISMA 2020 statement: an updated guideline for reporting systematic reviews. *BMJ* .2021;372: 10.1136/bmj.n71 n71 10.1136/bmj.n71 33782057PMC8005924

# Reviewer report: Reviewer response for version 1

**DOI:** 10.21956/openreseurope.16306.r30766

**Published:** 2023-02-20

**Authors:** Elena Mihajlova Stratilati

**Affiliations:** 1 Iustinianus Primus Law Faculty, Ss. Cyril and Methodius University in Skopje, Skopje, North Macedonia

Тhe text represents impressive research of what basically boils down to the basic question of how much and whether the need «to eliminate irregular border crossings of the external borders» – is in constant conflict with «a human rights-based approach to the European migrant policy ». The second sub-theme in this basic question is basically how much a security-focused, profit-driven, and technology-based approach to the problem is replacing one that prioritizes human rights, portraying migration as primarily a security threat and technology as the primary means to address it.

 The text makes in-depth analyzes to explore this perceived marginalization of human rights groups (HRGs) and civil society organizations (CSOs) in border surveillance, giving an account on the main reasons for it, as they are expressed by HRGs and CSOs. At the same time, this review takes into account and outlines the ways in which HRGs and CSOs are included in border management, focusing in particular on FRONTEX, from the point of view of legislation and of its consultancy bodies. In its conclusions, the paper introduces the ARESIBO Participatory Model (APM), a participatory methodology elaborated in the frame of the project ARESIBO as a proposal to enhance participation of HRGs and CSOs in the development of border surveillance technologies, thus addressing this perceived marginalization in a more consistent and structured manner. For this purpose, the paper is an application of extremely relevant knowledge about EU policies in the context of migration as well as the broader areas of migration and international human rights.

 Тhe paper abounds with references crucial to the research, but above all it is an extraordinary attempt to select relevant issues as reference points along which the discussion/elaboration moves and which make the research extremely relevant, together with a complete and detailed presentation of theses from "both sides of the argument".

 The thesis is an exceptional contribution to the field, relevant to all those who deal with migration, but also EU policies and human rights.

Are all factual statements correct and adequately supported by citations?

Yes

Is the review written in accessible language?

Yes

Are the conclusions drawn appropriate in the context of the current research literature?

Yes

Is the topic of the review discussed comprehensively in the context of the current literature?

Yes

Reviewer Expertise:

International law and relations, international human rights law, refugee law and migration

I confirm that I have read this submission and believe that I have an appropriate level of expertise to confirm that it is of an acceptable scientific standard.
